# A High-Protein Meal during a Night Shift Does Not Improve Postprandial Metabolic Response the Following Breakfast: A Randomized Crossover Study with Night Workers

**DOI:** 10.3390/nu12072071

**Published:** 2020-07-13

**Authors:** Nayara B. Cunha, Catarina M. Silva, Maria C. Mota, Caio A. Lima, Kely R. C. Teixeira, Thulio M. Cunha, Cibele A. Crispim

**Affiliations:** Graduate Programme of Health Sciences, Faculty of Medicine, Federal University of Uberlândia, Av. Pará, 1720, Bloco 2U, Campus Umuarama, Uberlândia 38405-320, Minas Gerais, Brazil; nayarabc8@hotmail.com (N.B.C.); catarinamsilva@yahoo.com.br (C.M.S.); carlam2006@yahoo.com.br (M.C.M.); caioaugustodelima@yahoo.com.br (C.A.L.); kelraspante@hotmail.com (K.R.C.T.); thcunha@yahoo.com.br (T.M.C.)

**Keywords:** night shift work, meal timing, high-protein meal, glucose tolerance, metabolic response

## Abstract

The aim of this study was to compare the acute effect of a high-protein/moderate carbohydrate (HP-MCHO) versus low-protein/high-carbohydrate (LP-HCHO) meal served at night on the postprandial metabolic response of male night workers the following breakfast. A randomized crossover study was performed with 14 male night workers (40.9 ± 8.9 years old; 29.1 ± 5.3 kg/m^2^). Participants underwent two different isocaloric dietary conditions at 1:00 h of the night shift: HP-MCHO (45 en% carbohydrate, 35 en% protein and 20 en% fat) and LP-HCHO (65 en% carbohydrate, 15 en% protein and 20 en% fat). Postprandial capillary glucose levels were determined immediately before the intake of the test meal and 30, 60, 90 and 120 min after the end of the meal. At the end of the work shift (6:30 h), participants received a standard breakfast and postprandial levels of glucose, insulin and triglycerides were determined immediately before and then every 30 min for 2 h (30, 60, 90 and 120 min). Higher values of capillary glucose were found after the LP-HCHO condition compared to the HP-MCHO condition (area under the curve (AUC) = 119.46 ± 1.49 mg/dL × min and 102.95 ± 1.28 mg/dL × min, respectively; *p* < 0.001). For the metabolic response to standard breakfast as the following meal, no significant differences in glucose, insulin, triglyceride, and HOMA-IR levels were found between interventions. A night meal with a higher percentage of protein and a lower percentage of carbohydrate led to minor postprandial glucose levels during the night shift but exerted no effect on the metabolic response of the following meal. This trial was registered at ClinicalTrials.gov as NCT03456219.

## 1. Introduction

Working patterns have changed due to economic and consumer demands, increasing the need for shift work with continued services 24 hours a day [1,2]. Shift work can be defined as any work schedule that is not performed on a regular daytime schedule (that is, approximately 07:00 to 18:00), and may include work in the early hours of the morning, evening or overnight [3]. Approximately 20% of the workforce in the world [4,5] and 10% in Brazil [6] are engaged in some sort of shift work schedule.

Studies have shown that this type of work is associated with a negative impact on health compared to conventional schedules, with a higher incidence of obesity and related diseases [7,8,9,10]. When compared with day workers, shift workers also present a higher prevalence and/or risk of diabetes mellitus [11,12], glucose intolerance [13], insulin resistance [14] and dyslipidemia [1]. Circadian misalignment [10] and lifestyle impairments, such as lower physical activity level [6], sleep deprivation [2,15] and eating habits [16,17], are postulated to be relevant causes in the development of these problems. From this perspective, inadequate food intake at night and the deleterious effect of this on the metabolism have been associated with the increased rates of metabolic disorders in these individuals [18].

Night food consumption is common among individuals who work at unconventional times [19]. In this sense, the quality, quantity and nutritional composition of meals consumed vary mainly because of the dependence on the availability of food preparations [20,21]. It is already known that night workers present more food events overnight, in a period when they should be sleeping, and the body is not metabolically prepared for food consumption [19]. However, although some studies point to negative impacts of nocturnal food intake on the metabolic response [18,22,23], the night worker usually eats meals at this time, which is influenced more by habit and time availability and less by appetite [21]. Thus, it is important that the literature tests possible nutritional strategies that provide energy and nutrient intake adequately for good professional performance and, at the same time, to minimize metabolic impairments.

A justification for the development of this study is that during the night, there is a reduction in glucose and lipid tolerance [13], which may have a prolonged effect the next day due to the sleep restriction imposed on the working condition [24]. In addition, it is known that there is a hierarchy in relation to the use of macronutrients by the human body, with the use of carbohydrates being a preferred energy source when compared to proteins and fats. However, carbohydrate intake leads to large metabolic changes, and these are exacerbated at night [25]. In contrast, protein intake has less of an effect on altering metabolism, mainly in plasma glucose, although this effect can occur due to the increase in postprandial insulin [26]. Thus, this lower stimulus of plasma glucose after eating dietary protein compared to eating carbohydrates during the night shift, which comprises an extended waking period, could lead to better results of the postprandial metabolic response the following morning, even with the presence of sleep restriction. The aim of this study was to test the acute effect of a high-protein versus low-protein meal served at night on the postprandial metabolic response of night workers the following breakfast. We hypothesize that a high-protein/moderate carbohydrate (HP-MCHO) meal during the night shift promotes a better postprandial metabolic response the following breakfast compared to a low-protein/high-carbohydrate (LP-HCHO) meal.

## 2. Methods

### 2.1. Participants and Ethics

This study was a controlled, randomized, crossover clinical trial conducted with fixed male night workers (nursing technicians, pharmacy technicians and administrative assistants) in a hospital in Uberlândia, Minas Gerais State, Brazil, between August 2017 and January 2018. They were recruited through phone calls and verbal communication. Eligible participants were males aged 20–50 years who were sedentary and working night shifts for at least 6 months. Exclusion criteria consisted of the inability to wear actigraphy monitors and carriers of diseases previously diagnosed and under treatment, such as type 2 diabetes mellitus, hypertension and related mood disorders (such as depression). All participants provided written informed consent. The study was approved by the Ethics Committee of the Federal University of Uberlândia on August 31st, 2017 (Protocol number 2,250,027) and was registered at ClinicalTrials.gov (NCT03456219).

To estimate the sample size, G*Power software version 3.1.9.2 (Heinrich-Heine-University Düsseldorf, Düsseldorf, Germany) was used for power analysis. Analysis of variance (ANOVA) test of repeated measures within and between interactions was selected with a statistical power (1–*β* error probability) of 0.95 and a moderately large effect size of 0.5 [27]. This estimation was used to detect differences between the means against the two phases of the study using an F test with an alpha level of 0.05. A minimum sample size of 10 participants was obtained. This sample size is similar to other studies that evaluated the postprandial metabolic profiles after meals and snacks eaten during simulated night and day work [18,28,29].

**Figure 1:**
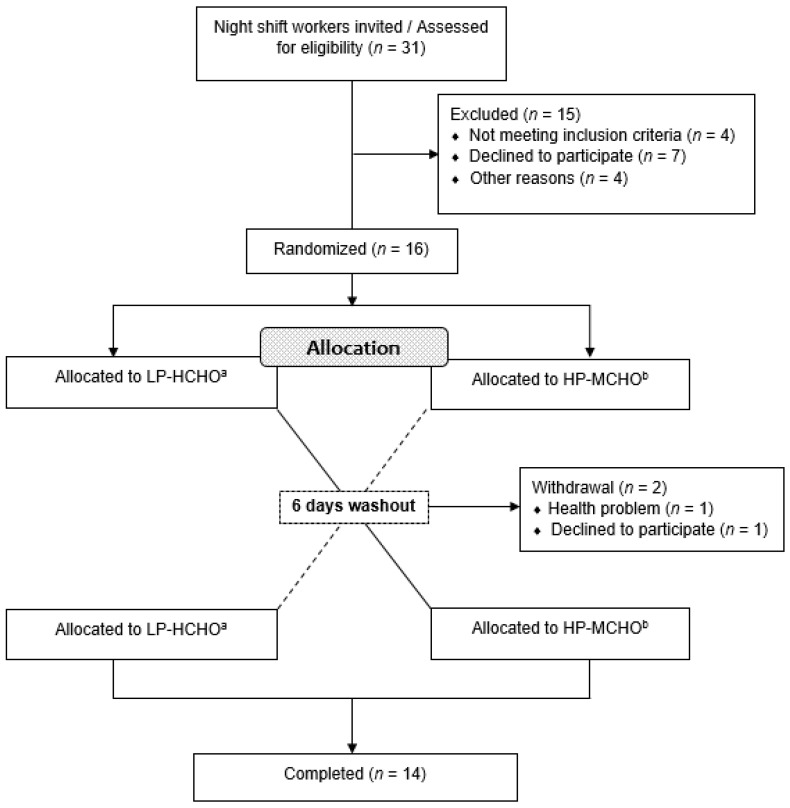
Summary of participant flow diagram. ^a^ Participants received a test meal composed of 65 en% carbohydrate, 15 en% protein and 20 en% fat. ^b^ Participants received a test meal composed of 45 en% carbohydrate, 35 en% protein and 20 en% fat. Abbreviations: HP-MCHO: High-protein/moderate carbohydrate; LP-HCHO, Low-protein/high-carbohydrate.

Thirty-one night shift workers were approached and invited to participate in the study while they were in the workplace. Before the invitation, a brief explanation of the research and procedures was given. During the study, seventeen participants were excluded or lost to follow-up or withdrew consent. Fourteen participants completed the trial. The study participant flow is shown in Figure 1.

### 2.2. Pre-Intervention Procedures

#### 2.2.1. Initial Questionnaire

All participants answered a questionnaire on personal information about sociodemographic characteristics, as well as sleep habits. For sleep duration and bedtime, participants were asked to report their usual bedtime, wake-up time, sleep-onset latency and usual sleep duration on workdays and free days. The questions used in the survey were: “What time have you been going to sleep on workdays during the past two weeks?”; “What time have you been waking up on workdays during the past two weeks?”; “What time have you been going to sleep on free days during the past two weeks?”; “What time have you been waking up on free days during the past two weeks?”; “How many minutes, on average, do you stay awake in bed before you fall asleep after turning the lights off?”. Sleep duration was computed using the weighted average of self-reported sleep duration, taking into consideration both workdays and free days [30].

#### 2.2.2. Anthropometric Evaluation

Weight was measured with a set of scales to an accuracy of 0.1 kg (Welmy W300). Height was measured with a stadiometer fixed to the wall, with an accuracy of 0.1 cm (Welmy W300). Both measurements were made according to the standards of Lohman et al. [31]. Body mass index (BMI, kg/m^2^) was calculated as the weight (kg) divided by the height squared (m^2^). Waist circumference (WC) was measured at the level of the umbilicus using an inextensible anthropometric tape (Sanny Medical, SN-4010, precision of 0.5 cm).

#### 2.2.3. Basal Metabolic Assessment

The blood samples used for performing the biochemical analyses were collected in Vacuette^®^ serum tubes by a nurse after an 8-h fasting period. The samples were centrifuged at 4000 rpm for 15 min at 4 °C and transferred immediately in cold boxes filled with ice to the laboratory. The serum levels of total cholesterol, high-density lipoprotein (HDL) cholesterol, low-density lipoprotein (LDL) cholesterol and triglycerides were evaluated by enzymatic colorimetry (Roche Diagnóstica©, São Paulo, Brazil), blood glucose levels by the hexokinase method (Roche Diagnóstica©, São Paulo, Brazil) and basal insulin by chemiluminescence (Roche Diagnóstica©, São Paulo, Brazil). Homeostatic model assessment for insulin resistance (HOMA-IR) was calculated using the formula: fasting insulin (mU/L) × fasting glucose (mmol/L)/22.5 [32]. Basal insulin and HOMA-IR values were considered by analyzing these parameters at time zero of the respective curves.

### 2.3. Experimental Protocol

**Figure 2:**
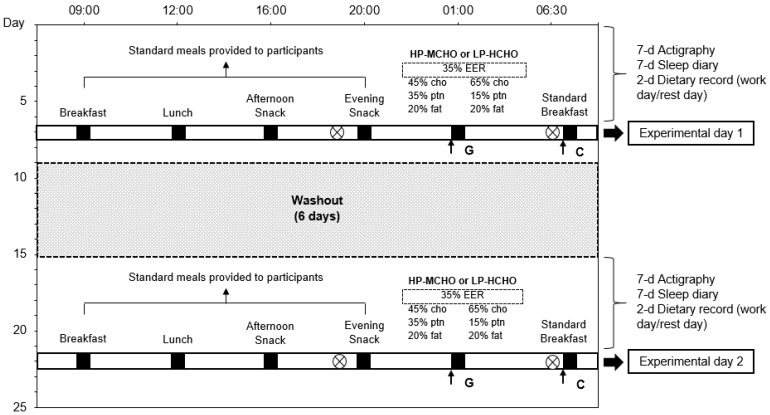
Protocol design of study. ⨂: Beginning and end of night shift (18:30 to 06:30). ■: Standard meals. **G**: Capillary glucose (0, 30, 60, 90, 120′). **C**: Curves glucose, insulin, triglycerides (0, 30, 60, 90, 120′). Abbreviations: HP-MCHO: High-protein/ moderate carbohydrate; LP-HCHO: Low-protein/high-carbohydrate; EER: Estimated Energy Requirement.

Participants were submitted to two different dietary conditions sorted randomly: HP-MCHO and LP-HCHO meals, with a 6-day washout period between them. Simple randomization was performed using the free resource Research Randomizer (http://www.randomizer.org). To ensure maintenance of the usual pattern of food intake and sleep in the experimental protocol, participants were followed-up for 7 days before each night intervention (run-in period) and evaluated in relation to their eating and sleep–wake habits using two food records (a work day and a rest day). The Sleep–wake pattern was monitored through a wrist actigraphy monitor (ActTrust, Condor) for 7 days in each condition. In addition, workers reported their sleep and wake times during the 7 days in a sleep diary. Study participants had a 12 h/36 h work schedule. Thus, the night before the seventh day (experimental day) they had one night off, that is, one night to sleep. Workers were instructed to eat at standardized times on the seventh day (09:00, 12:00, 16:00 and 20:00 h) and to eat everything in all meals provided by the research team (Table 1). No other foods were allowed during this day. The foods chosen for these meals, as well as the schedules, were those identified as usual among the volunteers. Energy requirement was calculated using the Harris–Benedict equation, with an activity level of 1.3 [22,33,34]. A summary of the study protocol is presented in Figure 2.

#### 2.3.1. Dietary Conditions

On the seventh day of follow-up two dietary conditions (HP-MCHO and LP-HCHO) were tested during the night work. A meal test was served at 01:00 h during the night work, which was performed from 18:30 to 06:30 h on the seventh day. The energy content of the meal test represented 30–35% of the estimated energy requirement (EER) of the day for each participant. The HP-MCHO intervention consisted of chicken, broccoli, carrot, lettuce, tomato, sugar-free orange juice and pineapple, comprising 45 energy (en%) carbohydrate, 35 en% protein and 20 en% fat. The LP-HCHO intervention consisted of pasta with tomato sauce, beef, carrot, lettuce, arugula, guava jam and orange juice with sugar, comprising 65 en% carbohydrate, 15 en% protein and 20 en% fat.

At the end of the night shift (06:30 h) for both conditions, participants received a standard breakfast with 65 en% carbohydrate (approximately 75 g), 10 en% protein and 25 en% fat, including potato bread, mozzarella cheese, wheat flour cake, papaya and orange juice with sugar. The nutritional composition of meals provided on experimental days is presented in Table 1. The amounts of energy, carbohydrate, protein and fat were calculated by Dietpro^®^ software (version 5.8.1, Agromídia Software^®^, Minas Gerais, Brazil) using the Brazilian Table of Food Composition [35]. If a component remained unidentified, the Food Composition Database of the United States Department of Agriculture (USDA) was used [36].

#### 2.3.2. Metabolic Assessment

Capillary glucose levels at night (01:00 h) were determined by glucometer (Accu-Chek^®^ Performa) immediately before eating the test meal (time 0) and 30, 60, 90 and 120 min after the end of the test meal. Blood samples used for performing biochemical analyses of plasma glucose, insulin and triglycerides the following breakfast were collected by a nurse in Vacuette^®^ serum tubes, obtained through an intravenous catheter immediately before (time 0) and every 30 min after the standard breakfast for 2 h (30, 60, 90 and 120 min). The samples were centrifuged within 1 h at 4000 rpm for 15 min at 4 °C and transferred immediately to the laboratory in cold boxes filled with ice. Serum levels of triglycerides were evaluated by enzymatic colorimetry (Roche Diagnóstica©, São Paulo, Brazil), blood glucose levels were evaluated by the hexokinase method (Roche Diagnóstica©, São Paulo, Brazil) and basal insulin levels were evaluated by chemiluminescence (Roche Diagnóstica©, São Paulo, Brazil). Homeostatic model assessment for insulin resistance (HOMA-IR) was calculated using the formula: fasting insulin (mU/L) × fasting glucose (mmol/L)/22.5 [32].

### 2.4. Statistical Analysis

Data normality was tested using the Shapiro–Wilk test. Results are represented as mean ± standard deviation (SD) for parametric data and as the median and interquartile range (25% and 75%) for non-parametric data. Comparison of sleep and dietary intake during the weeks before each protocol was performed using the Mann–Whitney U test. The generalized estimating equations (GEE) was used to analyze single and interaction effects of dietary intervention and time for the metabolic parameters (capillary glucose, plasma glucose, insulin, triglycerides and HOMA-IR), with adjustments for age, BMI, self-reported sleep duration and baseline metabolic parameters, and sequential Sidak correction post hoc. GEE are an extension of generalized linear models in that they allow adjusting for correlations between observations. Their robustness is related to the fact that they do not require the correct specification of the multivariate distribution, but only the average structure [37]. The area under the curve (AUC) with respect to the ground was calculated by the trapezoid method, and the difference between both interventions was analyzed using the generalized linear model (GzLM). Prism version 6.0 (GraphPad) was used for construction of the graphs (mean ± standard error of the mean) and for calculating the AUC. Statistical analysis was performed using SPSS version 21.0 software (IBM Corp., Armonk, NY, USA) and statistical significance was accepted as *p* <0.05 using the two-tailed test.

## 3. Results

The baseline characteristics of participants are presented in Table 2. Most participants were within the overweight range. Fasting plasma glucose was in the normal range of below 100 mg/dL [38]. Mean insulin and HOMA-IR levels were suggestive of hyperinsulinemia and insulin resistance, respectively. Mean values of total cholesterol (199.2 ± 48.6 mg/dL), LDL (127.1 ± 35.8 mg/dL) and triglycerides (157.0 (116.2–244.0) mg/dL) were above the recommended values, while mean HDL values (38.8 ± 7.5 mg/dL) were below the recommendation. Table 3 shows that participants did not differ in terms of nutritional and sleep characteristics between the two protocols.

The actigraphy and the sleep diary recorded naps during the day before the two experimental days (seventh day), which was not significantly different between the two protocols (experimental day 1 = 01:52 (00:00–4:07) hh:mm; experimental day 2 = 01:20 (00:00–2:25) hh:mm; *p* = 0.701).

**Figure 3:**
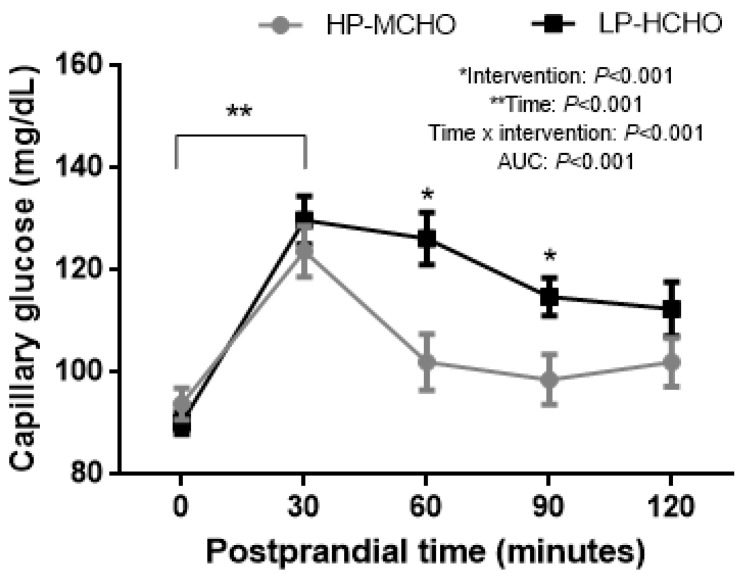
Capillary glucose curve after both interventions. Each curve represents the concentrations just before dinner (time 0) and 30, 60, 90 and 120 min after dinner. * Difference in glucose levels between the two conditions. ** Difference of time in both conditions. Data are represented as mean ± standard error of mean (SEM). Generalized estimating equation (GEE) was used to analyze the interaction between dietary intervention and time in the capillary glucose response, with adjustments for age, BMI, self-reported sleep duration and baseline metabolic parameters, and sequential Sidak correction post hoc (*p* value < 0.05).

Capillary glucose measured after the test meal showed lower values in the HP-MCHO condition compared to the LP-HCHO condition (AUC = 102.95 ± 1.28 and 119.46 ± 1.49 mg/dL × min, respectively; *p* < 0.001) (Figure 3). In addition, time and intervention interactions showed a significant effect, with higher values of glucose concentration over time in the LP-HCHO condition compared to the HP-MCHO condition. In the LP-HCHO condition there was a significant increase from time 0 to 30 min (90.16 ± 2.18 and 129.83 ± 4.73 mg/dL, respectively), remaining increased at 60 min (126.25 ± 5.05 mg/dL), 90 min (114.83 ± 3.68 mg/dL) and 120 min (112.41 ± 5.32 mg/dL); in the HP-MCHO condition there was a significant increase from time 0 (93.83 ± 3.05 mg/dL) to 30 min (123.75 ± 5.00 mg/dL), returning to baseline values at 60 min (102.00 ± 5.51 mg/dL) and remaining there up to 120 min (98.58 ± 4.91 and 102.00 ± 4.79 mg/dL; *p* < 0.001).

**Figure 4:**
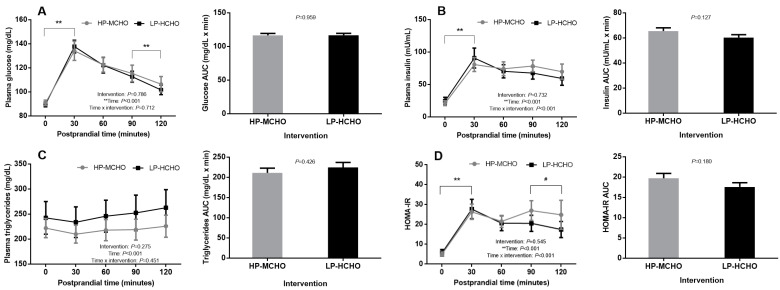
Curves and AUC of postprandial metabolic parameters. Each curve represents the concentrations just before standard breakfast (time 0) and 30, 60, 90 and 120 min after standard breakfast, of the following parameters: (**A**) Glucose; (**B**) Insulin; (**C**) Triglycerides and (**D**) HOMA-IR. ** Difference of time in both conditions. # Difference of time only in LP-HCHO condition. Data are represented as mean ± standard error of mean (SEM). Generalized estimating equation (GEE) was used to analyze the interaction between dietary intervention and time in the metabolic parameters, with adjustments for age, BMI, self-reported sleep duration and baseline metabolic parameters, and sequential Sidak correction post hoc (*p* value < 0.05).

Curves of postprandial metabolic parameters following breakfast are shown in Figure 4. There were no significant differences between the HP-MCHO and LP-HCHO conditions for tolerance (AUC = 116.53 ± 2.87 and 116.74 ± 2.87 mg/dL × min, respectively; *p* = 0.959) or plasma glucose concentration (*p* = 0.712). In both interventions there was a significant increase from time 0 (HP-MCHO: 90.91 ± 2.30 mg/dL; LP-HCHO: 89.66 ± 2.04 mg/dL) to 30 min (HP-MCHO: 134.25 ± 8.00 mg/dL; LP-HCHO: 137.75 ± 5.28 mg/dL) and a significant reduction from 90 min (HP-MCHO: 115.41 ± 6.86 mg/dL; LP-HCHO: 112.66 ± 4.59 mg/dL) to 120 min (HP-MCHO: 106.50 ± 6.35 mg/dL; LP-HCHO: 101.66 ± 3.89 mg/dL) (Figure 4A). Both meal tests had no effect on AUC (HP-MCHO: 65.56 ± 2.54 mU/mL × min; LP-HCHO: 60.28 ± 2.33 mU/mL × min; *p* = 0.127) or postprandial plasma insulin. In both interventions the postprandial insulin values increased from time 0 (HP-MCHO: 21.22 ± 3.79 mU/mL; LP-HCHO: 24.74 ± 5.58 mU/mL) to 30 min (HP-MCHO: 80.75 ± 10.74 mU/mL; LP-HCHO: 90.81 ± 15.38 mU/mL) and did not return to baseline values until 120 min (HP-MCHO: 69.85 ± 11.71 mU/mL; LP-HCHO: 59. 41 ± 10.65 mU/mL; *p* = 0.732) (Figure 4B). In the postprandial triglyceride curves there was no effect of the different interventions (HP-MCHO: AUC = 211.23 ± 11.55 mg/dL × min; LP-HCHO: AUC = 224.79 ± 12.29 mg/dL × min; *p* = 0.426); values remained unchanged (Figure 4C). Postprandial HOMA-IR also did not present any significant difference between interventions (HP-MCHO: AUC = 19.72 ± 1.18 x min; LP-HCHO: AUC = 17.57 ± 1.05 x min; *p* = 0.180) (Figure 4D). Despite this, there was a significant reduction from time 90 (20.60 ± 4.06) to 120 min (17.47 ± 4.06) in the LP-HCHO condition, which did not happen in the HP-MCHO condition (27.02 ± 4.97 and 24.90 ± 7.28).

## 4. Discussion

Studies conducted in non-shift workers have shown worse glucose tolerance after evening meals [18,23,39], which in theory would make high carbohydrate consumption deleterious at that time, possibly due to the deleterious effect of extended waking on glucose metabolism [40] and glucose tolerance [41], as well as the night effect of the endogenous circadian cycle on the glucose metabolism [42]. Different pathways could contribute to abnormal glucose metabolism linked to sleep restriction, such as reduced brain glucose utilization and insulin resistance [24]. However, in our study, eating a meal with higher protein content during the night shift, when compared to a high-carbohydrate meal, was not sufficient to minimize plasma glucose response and AUC in the standard breakfast after an extended wakefulness. As we expected, a higher carbohydrate intake at 01:00 h exacerbated the capillary glucose response compared to the HP-MCHO meal. Based on these findings, further research should evaluate the long-term metabolic effect of a HP-MCHO meal at night. In addition, protein intake, both in a single high-protein meal and in prolonged protein intake, has been shown to stimulate increased insulin secretion [43]; this hormone was not measured in our study at night after the test meal and should be measured in future studies.

Holmbäck et al. [44] examined the effect of high-carbohydrate (65% carbohydrate, 20% fat) and high-fat (40% carbohydrate, 45% fat) meals on the endocrine variables during a 24-h period (08:00, 12:00, 16:00, 20:00, 00:00 and 04:00 h). Similar to the present findings, the macronutrient composition of the evening meals had no impact on postprandial insulin response immediately after the meal compared to morning meals. However, it is important to highlight that our results in both interventions showed mean baseline insulin levels (time 0) close to the upper limit, according to the reference value (*Rv* = 2.60–24.90 mU/mL; LP-HCHO = 24.74 mU/mL vs. HP-MCHO = 21.22 mU/mL) [45]. In addition, insulin levels did not return to near basal values 2 h post-meal in both meals, suggesting insulin resistance in both conditions (LP-HCHO = 59.41 ± 10.65 mU/mL, *p* = 0.009; HP-MCHO = 69.85 ± 11.71 mU/mL, *p* = 0.001). The same was observed with HOMA-IR values: in both interventions, HOMA-IR values at time zero were higher than the reference values (*Rv* ≤2.7; LP-HCHO = 5.74 vs. HP-MCHO = 5.10) [45], which confirms insulin resistance. The insulin resistance present at night, which can be potentiated in chronically sleep-deprived individuals, such as night workers [14], reinforces that these individuals should not eat at night, or at least that the dietary strategies adopted should be able to minimize the metabolic losses of eating at a metabolically inappropriate schedule.

In our study, although the plasma triglyceride level presented no significant differences over the evaluation time in both interventions (Figure 4C), the level was above the reference value in both conditions (*Rv* fasting ≤150 mg/dL; *Rv* without fasting ≤175 mg/dL) [46]. Again, these results suggest that food consumption at night may have been improper in metabolic terms for both conditions and they affirm the previously demonstrated insulin resistance during nocturnal food intake since lipoprotein lipase is influenced by insulin; this plays a key regulatory role in postprandial triglyceride clearance [28,47]. Such a result is relevant for shift workers because elevated triglyceride concentrations are a strong and independent risk factor for ischaemic heart disease [48], and these individuals are more likely to have higher triglyceride concentrations [49], as observed in the baseline of the present study. In the Holmbäck et al. [50] study, the authors also observed higher triglyceride levels after consuming a high-fat diet at 05:00 and 06:00 h versus a high-carbohydrate diet and pointed out that this postprandial increase might be involved in the increased triglyceride concentrations seen in shift workers.

Some studies have proposed the strategy of not eating late at night as a way to prevent metabolic disorders [23,51,52] and even to favor professional performance [53]. In the Grant et al. [24] study, 11 males were divided into two groups in a clinical trial: eating at night (*n* = 4) or not eating at night (*n* = 7) over 4 nights of simulated night work. Glucose AUC in the eating at night condition was higher compared to not eating at night (969.0 ± 173.5 mmol/l vs. 815.5 ± 215.7 mmol/l, respectively; *p* < 0.001) in response to the meal tolerance test [23]. In the Sato et al. [29] study, ten individuals participated in a randomized repeated-measures design and the effect of a single loading of the late evening meal (22:30 h) compared to a normal evening meal (19:00 h) was evaluated on diurnal variation of blood glucose. Fasting blood glucose before breakfast the following day was similar in both dietary conditions (74 ± 2 vs. 77 ± 3 mg/dL, *p* = 0.231), but glucose AUC concentration during 5 h after breakfast was greater after late evening meal compared with that after normal evening meal (24,528 ± 844 vs. 26,291 ± 991 mg/dl min, *p* < 0.01). Despite this demonstrated impaired metabolic response, it is important to speculate about the motivations of shift workers in eating or not eating during a night shift. Waterhouse et al. [21] studied two groups, day workers and night workers, and evaluated by questionnaire those factors that influenced the type of food eaten. They found that the type and frequency of meals in the night workers were influenced significantly more by habit and time availability and less by appetite (*p* < 0.05) [21]. Night workers may want to eat food as a way to enjoy the pleasure of that intake or for social interaction in a period of rest. Nevertheless, a better nutritional option also needs to be established by considering together the metabolic aspects, habits and individual preferences.

The strength of this study lies in the clinical trial design performed in a population of night shift workers during their real work schedule and dietary control, performed the week before each protocol and on the experimental days. In addition, our study was one of the few that tested different meals at night as a strategy to meet nutritional needs during night work activities and to minimize the metabolic impact. The limitations of the study are related to the short time over which the interventions and evaluations were made. We did not include a group that did not eat at night, which limited the interpretation of our results. The inclusion of a prolonged fasting group and the evaluation of the effect of interventions on other parameters, such as glucagon, c-peptide and free fatty acids metabolism could provide a broader view on the topic. Also, regardless of the composition of the test meal, the fact that we controlled the energy and distribution of the macronutrients in a balanced way may have improved the response of these workers. In addition, only men were recruited for the study in order to reduce inter-individual variability and confounding factors, such as hormonal changes typical in women within the age range of the volunteers in this study, especially climacteric and menopause, which would have strongly influenced our outcome variables. However, the exclusion of female workers also limits the impact of the results of the present study and women need to be included in future studies in order to allow the generalization of these findings for shift workers of both sexes.

In conclusion, a night meal with a higher percentage of protein and a lower percentage of carbohydrates leads to lower postprandial glucose levels during the night shift but exerts no effect on the metabolic response the following meal. Further studies are necessary to assess the long-term metabolic response in this population.

## Figures and Tables

**Table 1:** Nutritional characteristics of meals provided to participants on experimental days.

	Energy (kcal)	Energy (kcal/kg)	Carbohydrate (g)	Carbohydrate (g/kg)	Carbohydrate (en%)	Protein (g)	Protein (g/kg)	Protein (en%)	Fat (g)	Fat (g/kg)	Fat (en%)
**Breakfast (09:00)**											
White bread, mozzarella cheese, whole milk and coffee with sugar	371.3 ± 61.9	4.3 ± 0.2	42.1 ± 7.0	0.5 ± 0.0	45.3 ± 0.0	16.5 ± 2.7	0.2 ± 0.0	17.8 ± 0.0	14.9 ± 2.5	0.1 ± 0.0	36.2 ± 0.0
**Lunch (12:00)**											
Rice, beans, beef, string bean, lettuce, tomato, raw carrot and pineapple juice with sugar	601.4 ± 100.3	6.9 ± 0.4	84.4 ± 14.1	1.0 ± 0.0	56.1 ± 0.0	40.7 ± 6.8	0.5 ± 0.0	27.1 ± 0.0	11.3 ± 1.9	0.1 ± 0.0	16.9 ± 0.0
**Afternoon Snack (16:00)**											
Strawberry yogurt, salt biscuit and banana	206.1 ± 34.4	2.4 ± 0.1	40.0 ± 6.7	0.5 ± 0.0	77.7 ± 0.0	4.6 ± 0.8	0.1 ± 0.0	8.9 ± 0.0	4.4 ± 0.7	0.1 ± 0.0	19.2 ± 0.0
**Evening Snack (20:00)**											
White bread, mozzarella cheese, ham, whole grape juice	432.0 ± 72.1	5.0 ± 0.3	65.8 ± 11.0	0.7 ± 0.0	60.9 ± 0.0	15.2 ± 2.5	0.2 ± 0.0	14.1 ± 0.0	11.8 ± 2.0	0.1 ± 0.0	24.6 ± 0.0
**Test meal HP-MCHO (01:00) - 35% EER**											
Chicken, broccoli, carrot, lettuce, tomato, sugar free orange juice and pineapple	753.2 ± 126.7	8.7 ± 0.5	83.0 ± 13.8	1.0 ± 0.0	44.1 ± 0.0	69.6 ± 11.6	0.8 ± 0.0	36.9 ± 0.0	16.1 ± 2.7	0.2 ± 0.0	19.2 ± 0.0
**Total intake of the day - HP-MCHO**	2364.0 ± 394.4	27.2 ± 1.5	315.3 ± 52.6	3.6 ± 0.2	53.3 ± 0.0	146.6 ± 24.5	1.7 ± 0.1	24.8 ± 0.0	58.5 ± 9.8	0.7 ± 0.0	22.3 ± 0.0
**Test meal LP-HCHO (01:00) - 35% EER**											
Pasta, tomato sauce, beef, raw carrot, lettuce, arugula, orange juice with sugar and guava jam	769.0 ± 128.3	8.8 ± 0.5	124.1 ± 20.7	1.4 ± 0.1	64.6 ± 0.0	28.8 ± 4.8	0.3 ± 0.0	15.0 ± 0.0	18.0 ± 3.0	0.2 ± 0.0	21.1 ± 0.0
**Total intake of the day - LP-HCHO**	2379.8 ± 397.0	27.4 ± 1.5	356.4 ± 59.5	4.1 ± 0.2	60.0 ± 0.0	105.8 ± 17.7	1.2 ± 0.1	17.8 ± 0.0	60.4 ± 10.1	0.7 ± 0.0	22.8 ± 0.0
**Standard breakfast**											
Potato bread, mozzarella cheese, wheat flour cake, papaya, orange juice with sugar	444.5 ± 0.0	5.3 ± 1.0	75.2 ± 0.0	0.9 ± 0.2	67.7 ± 0.0	11.6 ± 0.0	0.1 ± 0.0	10.4 ± 0.0	11.9 ± 0.0	0.1 ± 0.0	24.1 ± 0.0

Notes: Data are represented as mean ± SD. The meals breakfast, lunch, afternoon snack, and evening snack were identical in both conditions on the experimental days. The test meals (HP-MCHO and LP-HCHO), performed at 01:00, were identical in relation to the energy value, however, with different distribution of the macronutrients. The standard breakfast offered at the end of the night shift was identical in both conditions to perform the glucose, insulin and triglyceride curves. Abbreviations: HP-MCHO: High-protein/moderate carbohydrate; LP-HCHO: Low-protein/high-carbohydrate.

**Table 2:** Baseline characteristics of participants.

Variable	Night Shift Workers (*n* = 14)
**Demographic**	
Age (y) *	40.9 ± 8.9
**Anthropometry**	
Weight (kg) *	87.7 ± 18.8
Height (m) **	1.71 (1.69–1.77)
BMI (kg/m^2^) *	29.1 ± 5.3
Eutrophy % (*n*)	21.43 (3)
Overweight % (*n*)	42.85 (6)
Obesity % (*n*)	35.72 (5)
WC (cm) *	99.6 ± 10.8
**Sleep characteristics**	
Average sleep (h) *	5.5 ± 1.1
**Metabolic parameters**	
Glucose (mg/dL) **	81.5 (76–92)
Insulin (mU/mL) *	14.9 ± 7.9
HOMA-IR *	3.2 ± 1.8
Total cholesterol (mg/dL) *	199.2 ± 48.6
HDL (mg/dL) *	38.8 ± 7.5
LDL (mg/dL) *	127.1 ± 35.8
Triglycerides (mg/dL) **	157.0 (116.2–244.0)

Notes: * Mean ± standard deviation (SD) for parametric distribution; ** Median and interquartile range (25% and 75%) for non-parametric distribution.

**Table 3:** Comparison of sleep and dietary intake during the weeks before each protocol.

Variable	Night Shift Workers (*n* = 14)		
	Week before HP-MCHO	Week before LP-HCHO	*p* Value
Sleep duration (hh:mm)	06:52 ± 1:02	07:24 ± 1:20	0.248
Energy (kcal)	1816.68 (1472.66–2662.95)	2145.00 (1485.05–2482.72)	0.910
Energy (kcal/kg)	20.92 (15.76–31.82)	22.97 (17.72–29.07)	0.874
Carbohydrate (g/kg)	2.62 (1.93–3.99)	2.52 (1.88–3.35)	0.541
Carbohydrate (%)	49.31 (35.55–53.13)	45.09 (31.69–57.91)	0.769
Protein (g/kg)	1.19 (0.78–1.71)	1.21 (0.90–2.11)	0.769
Protein (%)	20.38 (17.42–26.29)	24.00 (19.16–28.58)	0.376
Fat (g/kg)	0.76 (0.44–1.17)	0.71 (0.38–1.12)	1.000
Fat (%)	30.75 (23.06–41.07)	28.63 (18.91–39.98)	0.769

Notes: Data are represented as mean ± SD for parametric distribution or median and interquartile range (25% and 75%) for non-parametric distribution. Abbreviations: HP-MCHO: High-protein/moderate carbohydrate; LP-HCHO: Low-protein/high-carbohydrate. Statistical comparison was performed by Mann–Whitney *U* test (*p* value < 0.05).

## References

[1] Canuto R., Garcez A.S., Olinto M.T.A. (2013). Metabolic syndrome and shift work: A systematic review. Sleep Med. Rev..

[2] Nea F.M., Kearney J., Livingstone M.B.E., Pourshahidi L.K., Corish A. (2015). Dietary and lifestyle habits and the associated health risks in shift workers. Nutr. Res. Rev..

[3] Bonham M.P., Bonnell E.K., Huggins C.E. (2016). Energy intake of shift workers compared to fixed day workers: A systematic review and meta-analysis. Chronobiol. Int..

[4] Barbadoro P., Santarelli L., Croce N., Bracci M., Vincitorio D., Prospero E., Minelli A. (2013). Rotating shift-work as an independent risk factor for overweight Italian workers: A cross-sectional study. PLoS ONE.

[5] Sun M., Feng W., Wang F., Li P., Li Z., Li M., Tse G., Vlaanderen J., Vermeulen R., Tse L.A. (2018). Meta-analysis on shift work and risks of specific obesity types. Obes. Rev..

[6] Alves M.S., Andrade R.Z., Silva G.C., Mota M.C., Resende S.G., Teixeira K.R., Gonçalves B.F., Crispim C.A. (2017). Social jetlag among night workers is negatively associated with the frequency of moderate or vigorous physical activity and with energy expenditure related to physical activity. J. Biol. Rhythm..

[7] Balieiro L.C.T., Rossato L.T., Waterhouse J., Paim S.L., Mota M.C., Crispim C.A. (2014). Nutritional status and eating habits of bus drivers during the day and night. Chronobiol. Int..

[8] Lowden A., Moreno C., Holmbäck U., Lennernäs M., Tucker P. (2010). Eating and shift work – effects on habits, metabolism and performance. Scand. J. Work Environ. Health.

[9] Liu Q., Shi J., Duan P., Liu B., Li T., Wang C., Li H., Yang T., Gan Y., Wang X. (2018). Is shift work associated with a higher risk of overweight or obesity? A systematic review of observational studies with meta-analysis. Int. J. Epidemiol..

[10] Vyas M.V., Garg A.X., Iansavichus A.V., Costella J.P., Donner A., Laugsand L.E., Janszky I., Mrkobrada M., Parraga G., Hackam D.G. (2012). Shift work and vascular events: Systematic review and meta-analysis. BMJ.

[11] Monk T.H., Buysse D.J. (2013). Exposure to shift work as a risk factor for diabetes. J. Biol. Rhythm..

[12] Vimalananda V.G., Palmer J.R., Gerlovin H., Wise L.A., Rosenzweig J.L., Rosenberg L. (2015). Night-shift and incident diabetes among African-American women. Diabetologia.

[13] Sharma A., Laurenti M.C., Man C.D., Varghese R.T., Cobelli C., Rizza R.A., Matveyenko A.V., Vella A. (2017). Glucose metabolism during rotational shift-work in healthcare workers. Diabetologia.

[14] Wright K.P., Bogan R.K., Wyatt J.K. (2013). Shift work and the assessment and management of shift work disorder (SWD). Sleep Med. Rev..

[15] Mota M.C., Waterhouse J., De-Souza D.A., Rossato L.T., Silva C.M., Araújo M.B.J., Tufik S., de Mello M.T., Crispim C.A. (2014). Sleep pattern is associated with adipokine levels and nutritional markers in resident physicians. Chronobiol. Int..

[16] Crispim C.A., Zimberg I.Z., Reis B.G., Diniz R.M., Tufik S., De-Mello M.T. (2011). Relationship between food intake and sleep pattern in health induviduals. J. Clin. Sleep Med..

[17] Mota M.C., De-Souza D.A., Rossato L.T., Silva C.M., Araújo M.B.J., Tufik S., de Mello M.T., Crispim C.A. (2013). Dietary patterns, metabolic markers and subjective sleep measures in resident physicians. Chronobiol. Int..

[18] Leung G.K.W., Huggins C.E., Bonham M.P. (2017). Effect of meal timing on postprandial glucose responses to a low glycemic index meal: A crossover trial in healthy volunteers. Clin. Nutr..

[19] Crispim C.A., Mota M.C. (2018). New perspectives on chrononutrition. Biol. Rhythm Res..

[20] Lowden A., Holmbäck U., Akerstedt T., Forslund A., Forslund J., Lennernäs M. (2001). Time of day type of food – relation to mood and hunger during 24 hours of constant conditions. J. Human Ergol..

[21] Waterhouse J., Buckley P., Edwards B., Reilly T. (2003). Measurement of, and some reasons for, differences in eating habits between night and day workers. Chronobiol. Int..

[22] Buxton O.M., Cain S.W., O’Connor S.P., Porter J.H., Duffy J.F., Wang W., Czeisler C.A., Shea S.A. (2012). Metabolic consequences in humans of prolonged sleep restriction combined with circadian disruption. Sci. Transl. Med..

[23] Grant C.L., Coates A.M., Dorrian J., Kennaway D.R., Wittert G.A., Heilbronn L.K., Pajcin M., della Vedova C., Gupta C.C., Banks S. (2017). Timing of food intake during simulated night shift impacts glucose metabolism: A controlled study. Chronobiol. Int..

[24] Reutrakul S., Van Cauter E. (2018). Sleep influences on obesity, insulin resistance, and risk of type 2 diabetes. Metab. Clin. Exp..

[25] Jebb S.A., Prentice A.M., Goldberg G.R., Murgatroyd P.R., Black A.E., Coward W.A. (1996). Changes in macronutrient balance during over- and underfeeding assessed by 12-d continuous whole-body calorimetry. Am. J. Clin. Nutr..

[26] Layman D.K., Shiue H., Sather C., Erickson D.J., Baum J. (2003). Increased dietary protein modifies glucose and insulin homeostasis in adult women during weight loss. Human Nutr. Metab..

[27] Cohen J. (1969). Statistical Power Analysis for the Behavioural Sciences.

[28] Al-Naimi S., Hampton S.M., Richard P., Tzung C., Morgan L.M. (2004). Postprandial metabolic profiles following meals and snacks eaten during simulated night and day shift work. Chronobiol. Int..

[29] Sato M., Nakamura K., Ogata H., Miyashita A., Nagasaka S., Omi N., Yamaguchi S., Hibi M., Umeda T., Nakaji S. (2011). Acute effect of late evening meal on diurnal variation of blood glucose and energy metabolism. Obes. Res. Clin. Pract..

[30] Reutrakul S., Hood M.M., Crowley S.J., Morgan M.K., Teodori M., Knutson K.L. (2014). The relationship between breakfast skipping, chronotype, and glycemic control in type 2 diabetes. Chronobiol. Int..

[31] Lohman T.G., Roche A.F., Martorrel R. (1988). Anthropometrics standardization reference manual. Medicine.

[32] Matthews D.R., Hosker J.P., Rudenski A.S., Naylor B.A., Treacher D.F., Turner R.C. (1985). Homeostasis model assessment: Insulin resistance and beta-cell function from fasting plasma glucose and insulin concentrations in man. Diabetologia.

[33] Harris J.A., Benedict F.G. (1918). A biometric study of human basal metabolism. Proc. Natl. Acad. Sci. USA.

[34] Cedernaes J., Brandell J., Ros O., Broman J.E., Hogenkamp O.S., Schiöth H.B., Benedict C. (2014). Increased impulsivity in response to food cues after sleep loss in healthy young men. Obesity.

[35] (2011). NEPA-UNICAMP—Núcleo de estudos e pesquisas em alimentação—NEPA/Universidade Estadual de Campinas—UNICAMP. Tabela Brasileira de Composição de Alimentos—TACO.

[36] US Department of Agriculture, Agricultural Research Service, Nutrient Data Laboratory USDA National Nutrient Database for Standard Reference, Release 28 (Slightly Revised). http://www.ars.usda.gov/ba/bhnrc/ndl.

[37] Ziegler A., Vens M. (2010). Generalized estimating equations: Notes on the choice of the working correlation matrix. Methods Inf. Med..

[38] American Diabetes Association (ADA) (2014). Diagnosis and classification of diabetes mellitus. Diabetes Care.

[39] Lopez-Mingues J., Saxena R., Bandín C., Scheer F.A., Garaulet M. (2018). Late dinner impairs glucose tolerance in MTNR1B risk allele carriers: A randomized, cross-over study. Clin. Nutr..

[40] Knutson K.L., Spiegel K., Penev P., Van Cauter E. (2007). The metabolic consequences of sleep deprivation. Sleep Med. Rev..

[41] Scheer F.A.J.L., Hilton M.F., Mantzoros C.S., Shes S.A. (2009). Adverse metabolic and cardiovascular consequences of circadian misalignment. Proc. Natl. Acad. Sci. USA.

[42] Spiegel K., Leproult R., Van Cauter E. (1999). Impact of sleep debt on metabolic and endocrine function. Lancet.

[43] Linn T., Santosa B.A.S., Groenemeyer D., Aygen S., Scholz N., Busch M., Bretzel R.G. (2000). Effect of long-term dietary protein intake on glucose metabolism in humans. Diabetologia.

[44] Holmbäck U., Forslund A., Lowden A., Forslund J.M., Åkerstedt T., Lennernäs M., Hambraeus L., Stridsberg M. (2003). Endocrine responses to nocturnal eating—Possible implications for night work. Eur. J. Nutr..

[45] Geloneze B., Vasques A.C.J., Stabe C.F.C., Pareja J.C., Rosado L.E.F.P.d., de Queiroz E.C., Tambascia M.A. (2009). HOMA1-IR and HOMA2-IR indexes in identifying insulin resistance and metabolic syndrome: Brazilian Metabolic Syndrome Study (BRAMS). Med. Arq. Bras. Endocrinol. Metabol..

[46] National Cholesterol Education Program (NCEP) Expert Panel on Detection, Evaluation, and Treatment of High Blood Cholesterol in Adults (Adult Treatment Panel III) (2002). Third report of the National Cholesterol Education Program (NCEP) expert panel on detection, evaluation, and treatment of high blood cholesterol in adults (Adult Treatment Panel III) final report. Circulation.

[47] Lund J., Arendt J., Hampton S.M., English J., Morgan L.M. (2001). Postprandial hormone and metabolic responses amongst shift workers in Antarctica. J. Endocrinol..

[48] Ginsberg H.N. (2001). Hypertriglyceridemia: New insights and new approaches to pharmacologic therapy. Am. J. Cardiol..

[49] Crispim C.A., Zalcman I., Dáttilo M., Padilha H.G., Edwards B.R., Waterhouse J., Tufik S., de Mello M.T. (2007). The influence of sleep and sleep loss upon food intake and metabolism. Nutr. Res. Rev..

[50] Holmbäck U., Forslund A., Forslund J.M., Hambraeus L., Lennernäs M., Lowden A., Stridsberg M., Åkerstedt T. (2002). Metabolic responses to nocturnal eating in men are affected by sources of dietary energy. Human Nutr. Metab..

[51] Barclay J.L., Husse J., Bode B., Naujokat N., Meyer-Kovac J., Schmid S.M., Lehnert H., Oster H. (2012). Circadian desynchrony promotes metabolic disruption in a mouse model of shiftwork. PLoS ONE.

[52] Sherman H., Genzer Y., Cohen R., Chapnik N., Madar Z., Froy O. (2012). Timed high-fat diet resets circadian metabolism and prevents obesity. FASEB J..

[53] Gupta C.C., Dorrian J., Grant C.L., Pajcin M., Coates A.M., Kennaway D.J., Wittert G.A., Heilbronn L.K., della Vedova C., Banks S. (2017). It’s not just what you eat but when: The impact of eating a meal during simulated shift work on driving performance. Chronobiol. Int..

